# High *WBP5* expression correlates with elevation of HOX genes levels and is associated with inferior survival in patients with acute myeloid leukaemia

**DOI:** 10.1038/s41598-020-60480-x

**Published:** 2020-02-26

**Authors:** C. Ward, P. Cauchy, P. Garcia, J. Frampton, M. A. Esteban, G. Volpe

**Affiliations:** 1 Joint School of Life Sciences, Guangzhou Medical University and Guangzhou Institutes of Biomedicine and Health, Guangzhou, 511436 China; 2 Laboratory of RNA, Chromatin and Human Disease, Guangzhou, 510530 China; 3 Key Laboratory of Regenerative Biology and Guangdong Provincial Key Laboratory of Stem Cell and Regenerative Medicine, Guangzhou Institutes of Biomedicine and Health, Chinese Academy of Sciences, Guangzhou, 510530 China; 4 Max Planck Institute of Immunobiology and Epigenetics, 79108 Freiburg, Germany; 5 Institute of Cancer and Genomic Sciences, College of Medical and Dental Sciences, University of Birmingham, B15 2TT Birmingham, UK; 6 Guangzhou Regenerative Medicine and Health Guangdong Laboratory, Guangzhou, 510005 China; 7 Institute for Stem Cells and Regeneration, Chinese Academy of Sciences, Beijing, 100101 China

**Keywords:** Acute myeloid leukaemia, Data mining

## Abstract

WW domain binding protein 5 (WBP5), also known as Transcriptional Elongation Factor A like 9 (TCEAL9) has been proposed as a candidate oncogene for human colorectal cancers with microsatellite instability and as a predictive indicator of small cell lung cancers. Furthermore, several independent studies have proposed *WBP5*, and its association with *Wilms Tumor-1* (*WT1*) expression, as part of a gene expression-based risk score for predicting survival and clinical outcome in patients with Acute Myeloid Leukaemia (AML). To date, the prognostic significance of the sole *WBP5* expression and its impact on the survival outcome in AML patients remains largely understudied. In the present study, we have made use of publicly available patient expression arrays and have developed an unbiased approach to classify AML patients into low versus high *WBP5* expressers and to balance them for known mutations and cytogenetic findings. Interestingly, we found that patients characterized by high *WBP5* expression displayed inferior overall and event-free survival rates. Notably, gene expression profiling showed that patients with high *WBP5* had elevated expression of several HOX cluster genes, such as *HOXA5*, *HOXA7*, *HOXA9* and *HOXA10*, and several of their partner proteins, such as *MEIS1* and *FOXC1*, which have been demonstrated to be causative for AML. Taken together, our data suggest that *WBP5* expression level could serve as an indicator for prognosis and survival outcome in patients with AML.

## Introduction

Acute myeloid leukaemia (AML) is a hierarchically-organized myeloproliferative disorder that is caused by stepwise acquisition of different mutations that prime malignant transformation and affect normal maturation of myeloid precursor cells^1,2^. Despite concerted efforts in the development of new treatments, many patients are refractory to current therapeutic approaches or have a high relapse rate, with the overall long-term survival of patients being below 40% and more than 60% of the patients over 65 years of age succumbing to the disease within one year of diagnosis^3^. In current medical practice, the diagnosis, prognosis, and therapeutic choices are dictated by detection of genetic mutations and the measurement of specific biomarkers that are used to classify patients into risk categories. However, due to the heterogeneous nature of the disease, prognosis within these categories is highly variable.

Aside from chromosome lesions, such as those involving MLL (i.e. MLL-AF9, MLL-ENL)^4,5^ or RUNX1 (i.e. RUNX1-ETO, RUNX1-EVI1)^6,7^ translocations, common prognostic and categorization factors involve mutations in the genes encoding signalling proteins (FLT3, RAS and KIT)^8,9^, transcription factors (CEBPA, PU.1 and GATA2)^10–12^ and DNA methylation related genes (TET2, DNMT3A, IDH1 and IDH2)^13–16^. Patient stratification has been further refined by the advent of next-generation whole-genome and transcriptome sequencing technologies; however, the identification of new reliable biomarkers is still required in clinical practice for use as prognostic factors and as new actionable therapeutic targets.

WW domain binding protein 5 (*WBP5*), a novel upstream regulator of the Hippo pathway^17^, has been recently associated with a variety of cancers, such as advanced gastric cancer with aggressive lymph node metastasis^18^, colorectal cancers with microsatellite instability^19^ and in small cell lung cancers^20^ where it has been reported to influence tumour growth by promoting cell proliferation and inhibiting apoptosis. A link of *WBP5* with leukaemia has become evident in recent years through the generation of gene expression prognostic signatures for predicting clinical outcomes in patients with AML. In fact, by making use of cDNA microarrays, Metzeler and co-workers presented *WBP5* as part of a gene expression-based signature that comprises an 86-probe set (66 genes), which was used for predicting survival outcome in patients with cytogenetically normal AML (CN-AML)^21^. Subsequently, Bou Samra *et al*. developed an independent and further refined gene expression-based risk score in which *WBP5* was part of a 22-gene signature that displayed a strong prognostic value in 2 independent cohorts of CN-AML patients^22^. Recently, Niavarani *et al*. have reported *WBP5* as part of a 17-probe set signature to predict unfavourable outcome in association with high levels of *WT1* in AML patients^23^. To date, whether the sole expression of *WBP5* could serve as a prognostic indicator and whether its expression has any impact on the establishment and maintenance of myeloid diseases has not been assessed.

In the present study, we have taken an unbiased bioinformatic approach to identify new molecular biomarkers by making use of publicly available patient gene expression arrays in which the whole AML patient cohort was ranked according to the expression of every gene probe into high and low expressers and used to determine the impact of this classification on the overall and event-free survival outcome and on the gene expression profiles. Our approach identified *WBP5* as one of the most significant genes and showed that high expression of *WBP5* is associated with a markedly inferior outcome and with an elevation of leukaemia associated HOX gene clusters expression. The prognostic value of *WBP5* was validated in five independent AML gene expression datasets, thus suggesting *WBP5* to be a new reliable molecular biomarker and a new potential therapeutic candidate for AML patients.

## Materials and Methods

### Patient profiling arrays information

The overall survival (OS) and event-free survival (EFS) scores were determined using non-parametric Kaplan-Meyer estimates; comparison of survival between the low and high WBP5 subgroups was based on two-sided log rank test.

### Data processing

GSE6891^24^, GSE15434^25^, GSE13204^26^, GSE1159^27^, GSE22845^28^ microarray raw data were downloaded from NCBI Gene Expression Omnibus (GEO). For each probe in each dataset, expression was scaled from 0 to 1. Then high and low expressing patient groups were established using 0.7–1 or 0–0.3 expression, respectively. Once subgroups were determined, raw CEL data was downloaded for each patient and expression values were calculated, background corrected, log2 transformed and quantile normalized in R (version 3.6.0) using affy package (version 1.62.0) and the rma function. Differential gene expression was carried out using the limma package (version 3.40.2) by fitting a linear model of high vs low patients for WBP5 expression.

### Subgroup balancing

After selecting high and low expressing patients as separate subgroups, we checked if there was a significant proportion of each subgroup that contained patients with specific cytogenetic abnormality, disease marker expression, age or gender using Fisher exact test. If we found a significant imbalance, we adopted a randomization strategy to balance the groups. Patients were shuffled in and out of the high and low expressing subgroups until there was no significant proportion of a specific cytogenetic abnormality, disease marker expression, age or gender.

### Survival analysis

For the GSE6891, GSE12417^21^ and GSE37642^29^ datasets, the high expressing subgroup and the low expressing subgroup were compared for each probe using overall survival data. Event-free survival was analysed for GSE6891 dataset only. A p-value was calculated to determine significant differences using Wilcoxon rank-sum test.

Kaplan-Meier plots were generated using Python (version 3.5.5) lifelines package (version 0.14.6). P-values represent Wilcoxon rank-sum test results comparing high and low expressing patients.

### Unsupervised hierarchical clustering

The hierarchical clustering of patients shown in this study was unsupervised and was performed on normalized data using Pearson correlation Euclidian distance metric with complete linkage agglomeration method. Hierarchical clustering of genes was done using the z-score values of the genes based on Euclidian distance metric with complete linkage agglomeration method.

### Statistical analysis

Statistical analysis throughout this study was determined by performing t-test for pairwise comparison and the p-values are indicated where appropriate.

## Results

### Generation of a workflow for identification of new molecular biomarkers

**Figure 1:**
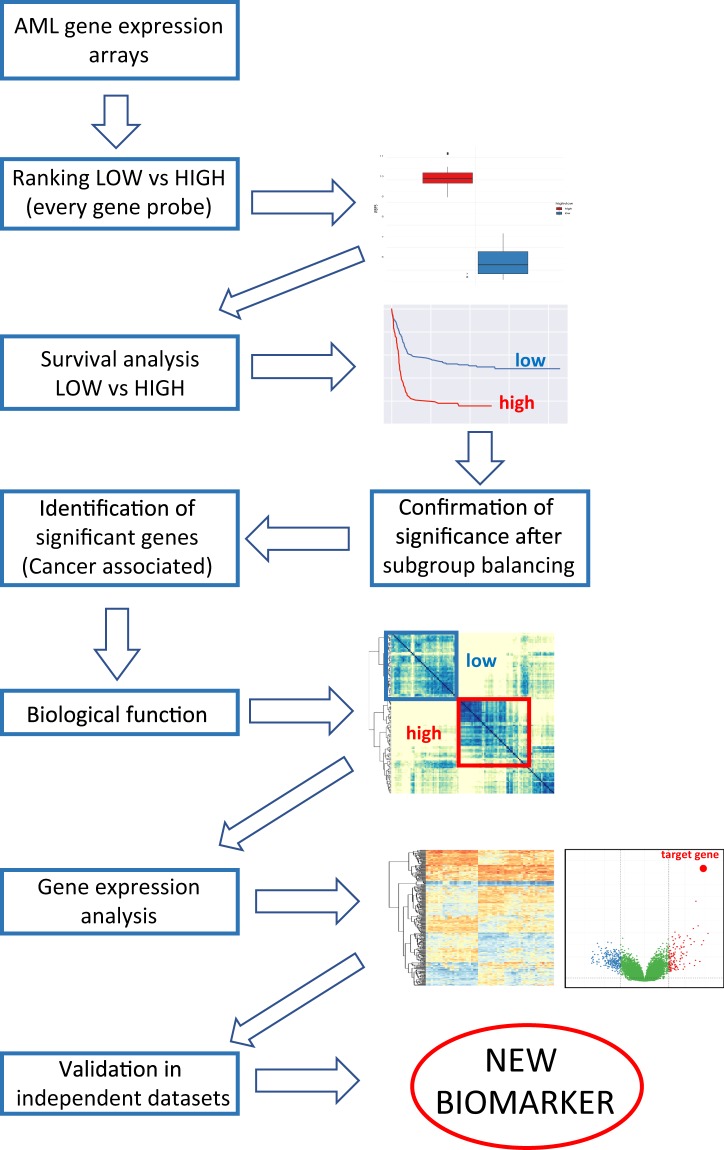
Flow chart showing the sequential steps for the identification of new molecular biomarkers from AML patient expression arrays.

To search for new potential molecular biomarkers for AML, we retrieved 5 independent publicly available microarray datasets GSE6891^24^ (461 samples), GSE15434^25^ (251 samples), GSE13204^26^ (542 samples), GSE1159^27^ (285 samples) and GSE22845^28^ (154 samples) from the Gene Expression Omnibus (GEO) public repository. Firstly, we applied our pipeline described in Fig. 1 to the GSE6891 dataset as it encompasses patients with different types of AML and every patient within the cohort is annotated with a comprehensive mutational and survival analysis. We developed a computer algorithm to systematically screen every gene probe within the dataset by ranking all patients from the lowest to the highest and classifying them into low (0–30% of the expression range) and high expressers (70–100%) for each given probe. For each gene, a comparison between the low and high subgroups was then made by screening survival data to identify those that have a statistically significant impact on both overall and event-free survival, i.e. those demonstrating a p-value lower than 0.05 as calculated by Wilcoxon rank-sum test. Upon identification of a positive hit, low and high expresser subgroups were adjusted for age, sex, cytogenetic findings, and mutational status and the survival analysis was repeated. After balancing, the list of genes that demonstrated a significant survival impact was then trimmed based on the involvement or deregulation of those genes in cancer; the genes that were positive by all those criteria were then surveyed for their influence on global gene expression and further validated in the other independent datasets. *WBP5*, a gene that has recently been associated with a variety of cancers and haematological malignancies, passed all criteria and became the main subject of this study.

### High *WBP5* expression is associated with inferior survival in AML patients

**Figure 2:**
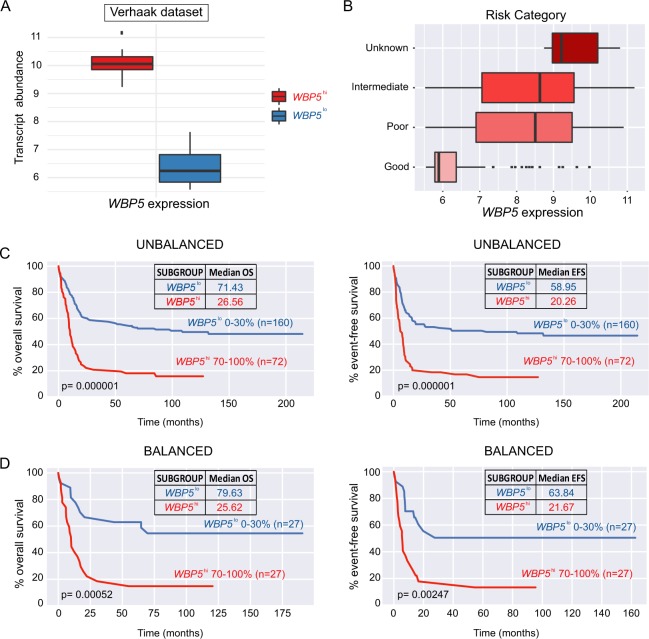
High *WBP5* expression is associated with unfavourable outcome in patients with AML. (**A)** Boxplot representation of *WBP5* expression boundaries for patient samples from the Verhaak *et al*. dataset (0–30% low expression, 70–100% high expression). **(B)** Boxplot depicting the association of *WBP5* expression with risk group categorization (poor, intermediate, good and unknown). **(C)** Kaplan-Meier representation of overall (OS) and event-free (EFS) survival for *WBP5*^*low*^ and *WBP5*^*high*^ AML patients prior to or **(D)** after balancing for age, sex, FAB and cytogenetic findings. The number of patients and the median OS and EFS are indicated in the plot. P-value was calculated using Wilcoxon rank-sum test.

In the patient cohort described by Verhaak *et al*.^24^ we ranked patients according to their *WBP5* expression levels and selected the bottom 30% of the whole expression range as low expressers (*WBP5*^*low*^, n = 160) and the top 30% as high expressers (*WBP5*^*high*^, n = 72) as indicated in Fig. 2A. We found a large association of high *WBP5* levels with poor cytogenetic risk, while a large proportion of low expressers belonged to the good risk subgroup (Fig. 2B). Next, we compared the survival outcomes for the whole cohort and observed *WBP5*^*high*^ patients to be associated with a significantly unfavourable overall survival (median OS = 26.56 vs 71.43, p = 0.000001) and event-free survival outcome (median EFS = 20.26 vs 58.95, p = 0.00001) (Fig. 2C).

Furthermore, we tested how *WBP5* expression could be modulated in different AML subtypes by analysing the mutational status of the two subgroups (*WBP5*^*high*^ vs *WBP5*^*low*^); this approach demonstrated a correlation of high *WBP5* expression with adverse cytogenetic findings such as NPM1c and FLT3-ITD, these being found in 43% vs 15% (p = 0.0000089) and in 40.2% vs 13.7% (p = 0.000013), respectively. Moreover, we found EVI1 to be more frequent in the *WBP5*^*high*^ group when compared to the *WBP5*^*low*^ group (15.27% vs 1.25%, p = 0.000063); conversely, CEBPA mutations, that are normally associated with favourable prognosis, were exclusively found in *WBP5*^*low*^ patients (11.25% vs 0%, p = 0.0011). Furthermore, we observed the low expressers to be largely associated with idt(16) (14.37% vs 1.38%, p = 0.0018) and t(8;21) translocation (22% vs 0%, p = 0.0000046), a genetic lesion normally associated with good overall survival. No relationship between *WBP5* expression and age or sex was observed (Table 1).

**Table 1:** Genetic and karyotypic characteristics of the *WBP5*^high^ and *WBP5*^low^ patient subgroups.

	WBP5^low^ (n = 160)	WBP5^hi^ (n = 72)	p-value
Molecular abnormalities			
IDH1	7	4	0.74231192
IDH2	5	6	0.10035424
NPM1c	24	31	8.95E-06
FLT3-ITD	22	29	1.38E-05
FLT3-TKD	17	7	1
NRAS	22	4	0.07474453
KRAS	4	0	0.31345029
EVI1	2	11	6.37E-05
CEBPA	18	0	0.00112984
Karyotypic abnormalities			
+8	10	4	1
−5/7q	6	10	0.00925701
−9q	5	0	0.32772068
11q23	5	3	0.70598866
complex	8	0	0.06052875
NN	50	31	0.10135058
Other	14	9	0.47627351
abn(3q)	0	1	1
failure	1	4	0.03330118
idt(16)	23	1	0.00180154
t(15;17)	1	0	0.1433
t(6;9)	0	1	0.31034483
t(8;21)	32	0	4.68E-06
t(9;22)	1	0	1
FAB classification			
M0	8	3	1
M1	25	27	0.00054777
M2	39	20	0.62606826
M4	36	8	0.04664368
M5	39	8	0.0216892
M6	0	1	0.31034483
unknown	3	0	0.55419693
Sex			
Male	80	31	0.39421991
Female	80	41	0.39421991

The table shows data for the 232 patients from the Verhaak dataset (GSE6891), including the occurrence of common karyotypic lesions and molecular aberrations, FAB classification and sex of patients.

Considering that NPM1c and FLT3-ITD, often found together, are indicators of bad prognosis while CEBPA and t(8;21) predict a more favourable outcome, we postulated that this observation could account for the inferior overall and event-free survival observed in the cohort of *WBP5*^*high*^ patients. To rule out this possibility, the subgroups of low and high expressers were adjusted for age, cytogenetics findings, FAB classification and mutational status to avoid a potential bias to a specific driver mutation or subtype.

Surprisingly, survival analysis after balancing revealed that *WBP5*^*high*^ patients (n = 27) still displayed inferior overall (median OS = 25.62 vs 79.63, p = 0.00052) and event-free survival (median EFS = 21.67 vs 63.84, p = 0.00247) when compared to *WBP5*^*low*^ cohort (n = 27), thus suggesting *WBP5* expression to be a strong disease indicator independently of its association with specific oncogenic mutations. *WBP5* was also reported in three independent studies to be part of gene expression risk scores that predict adverse outcomes in AML patients;^21–23^ to further determine the validity of *WBP5* as a potential biomarker and to assess whether *WBP5* expression alone could predict the inferior outcome determined in those cohorts by the use of those risk panels, we retrieved those datasets and performed survival analysis comparing *WBP5* low and high expressers. In agreement with the results from our pipeline, high expressers demonstrated lower survival rates in both cohorts after balancing (median OS = 347.25 vs 691.14, p = 0.01621 and 496.33 vs 1180.73, p = 0.01478) (Fig. S1), thus suggesting that the sole expression of *WBP5* could serve as a reliable inferior predictor for AML.

### *WBP5*^*high*^ AML patients display a specific gene expression profile

**Figure 3:**
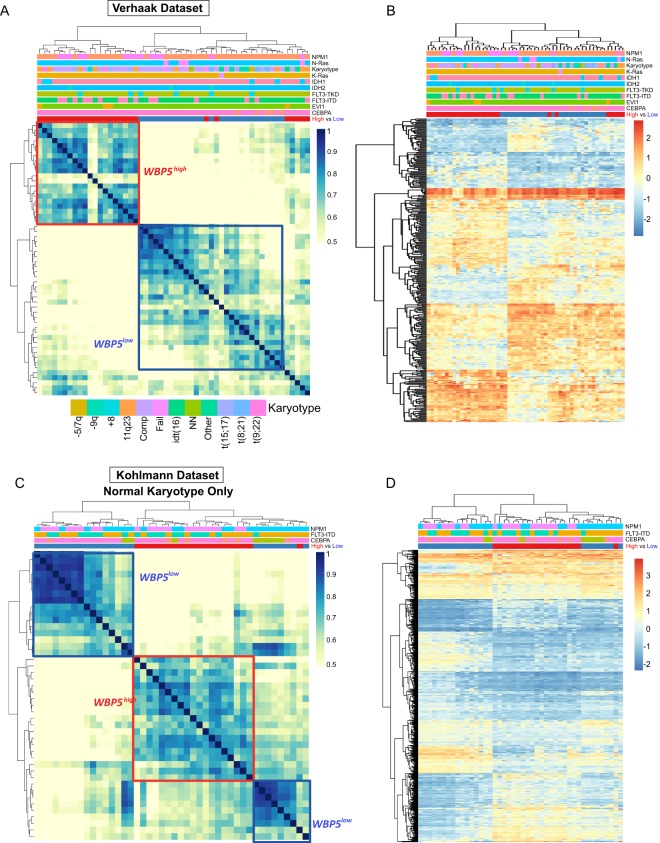
*WBP5*^*high*^ and *WBP5*^*low*^ subgroups have distinct global gene expression profiles. (**A)** Hierarchical clustering of the Pearson correlation coefficient of mRNA expression showing distinct clusters for high and low *WBP5* expressers from the Verhaak cohort. The mutational status of every patient is indicated by a colour coded graph on the top of the clustering map. A legend indicating each genetic subgroup is placed below the clustering map. **(B)** Heatmap indicating the gene expression profiles of *WBP5*^*low*^ and *WBP5*^*high*^ subgroups. **(C)** Hierarchical clustering of the Pearson correlation coefficient and **(D)** heatmap of mRNA values from the *WBP5*^*low*^ and *WBP5*^*high*^ subgroups of the patient cohort reported by Kohlmann *et al*. The cluster of low and high *WBP5* expressers are indicated with blue and red boxes, respectively.

In order to investigate the molecular profiles associated with different levels of *WBP5*, we sought to identify similarities and differences between the low and high *WBP5* expressers by performing differential gene expression analysis between the two groups. Differentially expressed genes were considered as those displaying a log fold change (FC) >1 and an adjusted p-value < 0.05. These genes were used to hierarchically cluster the patients using Pearson correlation coefficient. This analysis showed that most of the high expressers (20 out of 27 patients) formed a discrete cluster with high correlation coefficient, with the exception of a subgroup of 5 *WBP5*^*high*^ patients that clustered separately and more closely with the low *WBP5* cohort (Fig. 3A). This could be due to those patients being characterized by a specific mutation that was not screened for this analysis.

Next, we studied the identified differential genes that are specifically deregulated in the *WBP5*^*high*^ subgroup and observed that the high expresser subgroup displayed a distinct gene expression profile, as indicated in the heatmap in Fig. 3B.

To validate our observations, we made use of another independent gene expression profiling dataset reported by Kohlmann *et al*.^25^. For this dataset, which comprises 251 AML patients with normal karyotype, we applied the same classification criterion for ranking them into high (n = 23) and low *WBP5* expressers (n = 21) and balancing for sex, age, FAB and cytogenetic findings. Although this cohort does not encompass patients carrying karyotypic lesions, our analysis showed a similar trend, with most of the high expressers forming a defined cluster (Fig. 3C). Moreover, those patients again displayed a distinct gene expression profile compared to the low expressers as previously seen for the Verhaak dataset, thus strengthening the validity of our observations (Fig. 3D). We further confirmed this trend in three other independent cohorts, such as those reported by Valk *et al*.^27^, Haferlach *et al*.^26^ and Taskesen *et al*.^28^. Similarly, in the Valk and Taskesen datasets we observed that those patient subgroups formed moderate clusters with good correlation coefficients, with the exception of the Haferlach dataset in which, despite clustering together, both high and low expressers seemed to form rather smaller sub-clusters (Fig. 2), possibly due to the fact that those patients could not be balanced for the lack of information on their cytogenetic and mutational statuses.

### High *WBP5* expression is associated with an elevated HOX cluster gene expression

**Figure 4:**
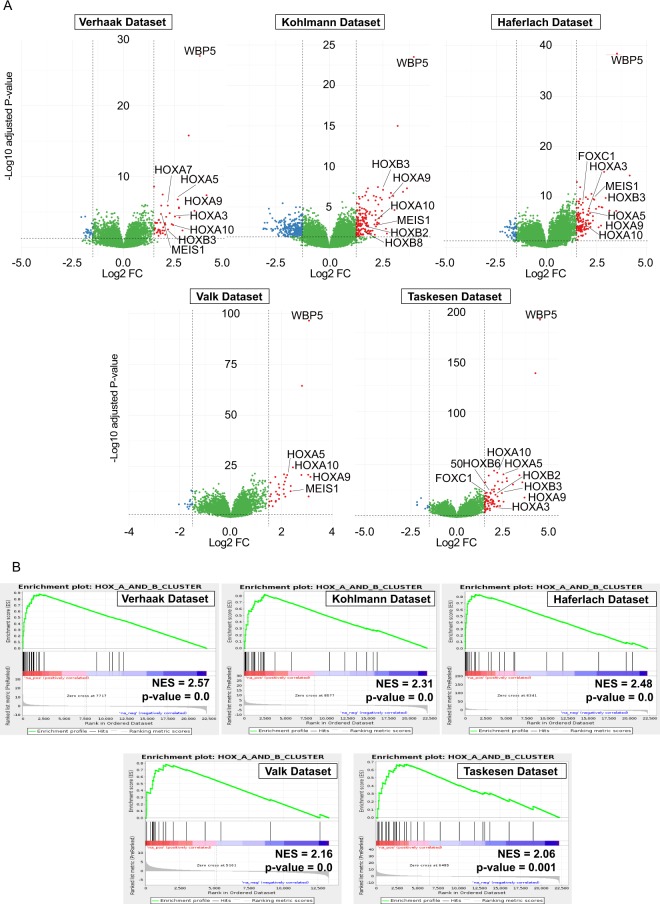
Correlation of high *WBP5* expression with HOXA and HOXB clusters gene signatures. (**A)** Volcano plots indicating the most differentially more highly (red dots, above 1.5 log2 FC) and lowly expressed genes (blue dots, below −1.5 log2 FC) in the GSE6891 (Verhaak), GSE15434 (Kohlmann), GSE13204 (Haferlach), GSE1159 (Valk) and GSE22845 (Taskesen) datasets. The most highly differentially expressed HOXA, HOXB and HOX-TALE genes are indicated in each plot. **(B)** Gene Set Enrichment Analysis (GSEA) plots show enrichment of HOXA and HOXB clusters genes ranked using a signal-to-noise metric according to expression in *WBP5* low versus high expressers.

By performing differential gene expression analysis on all the five independent datasets, within the core of the high *WBP5* expressers we considered as more highly expressed genes with an average log2 fold change value of at least 1.5 above and downregulated genes with values at least 1.5 below that of low expressers. Note that in the 15 genes showing highest differential expression in most of the cohorts (above 2 log2 FC) we observed several members of the HOXA and HOXB gene clusters, among which *HOXA9*, *HOXA5* (with the exception of Kohlmann dataset), *HOXA10*, *HOXB2* and *HOXB3* (Fig. 4A,B). The full expression data for all the HOXA and HOXB cluster genes are provided in Supplementary Fig. 3. Intriguingly, several genes that are known partners of *HOXA9*, such as *MEIS1*, were also found to be differentially expressed. This has profound implications as those genes play key roles in the progression and maintenance of different types of myeloid diseases, and the combination of *HOXA9* and *MEIS1* has been shown to be causal for the development of AML^30–32^.

We also observed a moderate correlation of *WBP5* expression with the levels of *HOXA7*, another gene that was reported to influence leukaemia latency and phenotype and is required for efficient immortalization of myeloid cells by MLL-ENL fusions^33^. Analysis of the HOX-Tale partner proteins also revealed higher levels of *PBX3* transcript in all cohorts, but not that of *PBX1* or *PBX2*.

This analysis also pointed to the higher expression of several other genes normally deregulated in a wide spectrum of leukaemias, including genes that act as transcriptional regulators (*PRDM16* and *NFIX*) and a gene that influences proliferative advantage and survival (*IGFBP2*). Moreover, we observed higher expression of a number of genes that are part of the FLT3-ITD molecular signature, such as *HOXB2*, *HOXB3*, *HOXB4*, *HOXB5*, *HOXB6*, *CRNDE*, *CLU*, *CTSG*, *COL4A5* and *KRT18*, among others (Fig. S4)^34,35^.

**Figure 5:**
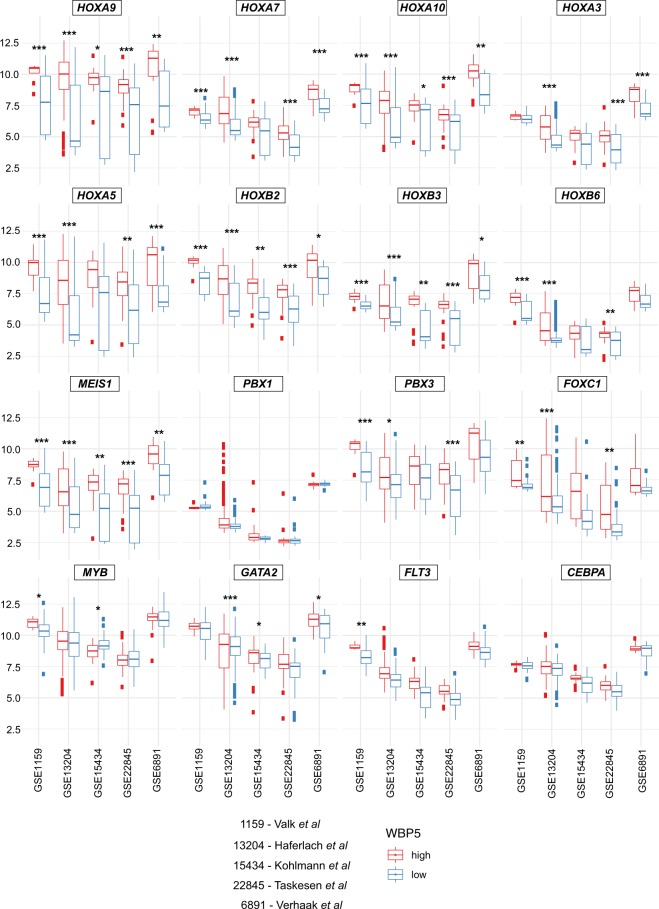
Elevated expression of HOXA, HOXB and HOX partner genes across five different independent gene expression datasets. Expression of representative HOXA (*HOXA9*, *HOXA7*, *HOAX10* and *HOXA5*) and HOXB (*HOXB2*, *HOXB3*, *HOXB6*) cluster genes and HOX partners (*MEIS1*, *PBX1*, *PBX3* and *FOXC1*) or downstream effectors (*MYB*, *GATA2*, *FLT3* and *CEBPA*). Data are represented as boxplots in which high expressers are indicated in red and low expressers are indicated in blue. Every plot presents a color-coded boxplot showing median and interquartile ranges. The statistical analysis reported indicates p-value adjusted for false discovery rates. ***<0.001, **<0.01, *<0.05.

We also looked at the expression of genes that have been shown to be directly involved in HOXA-driven leukemogenesis and observed a significant increase in the expression of *GATA2*^36^, while no difference was observed for *FLT3*^9,37,38^, *MYB*^39,40^ or *CEBPA*^41^. Intriguingly, we also noted increased expression of *FOXC1*^42^, although in this cohort it failed to reach statistical significance (Fig. 5).

To confirm these observations, we calculated the average mRNA levels of *WBP5*^*high*^ and *WBP5*^*low*^ patients from all the other independent patient gene expression datasets; notably, we observed a gene expression pattern similar to the one obtained from the Verhaak cohort, that is, a large elevation of several HOXA and HOXB cluster members, such *HOXA9*, *HOXA10* and HOX-TALE partner *MEIS1* (Figs. 4A,B, 5 and S3). Several genes which influence gene expression, cell growth or that are part of FLT3-ITD signature were confirmed in virtually all cohorts as more highly expressed genes, including *CPA3*, *PRDM16*, *IGFBP2* and *CTSG* (Fig. S4).

In a recent study by Naivarani *et al*., *WBP5* was included in a 17-probe set signature that is used as predictor of long-term prognosis in AML patients characterized by high *WT1* levels^23^. To test this, we probed for this relationship in our pipeline and observed a strong association between *WBP5* and *WT1* transcripts in all the dataset tested. (Fig. S3).

In summary, these analyses highlight *WBP5* as a reliable prognostic and stratification biomarker and the association between high *WBP5* expression and the elevation of HOX cluster genes levels.

## Discussion

AML is among the most malignant cancers of the blood and due to its heterogeneous nature and complex biological behaviours there are currently limited therapeutic approaches.

The ability to diagnose and prevent myeloid leukaemias is hampered by the lack of quantifiable, reliable, and easily measurable biomarkers that correlate with disease progression. Steady improvements in survival rate and disease control have been made in the past decades, but despite the efforts aimed at developing new personalized and sensitive therapeutic approaches for patient therapy, AML is still associated with high morbidity and mortality. Recent advances in transcriptomic and epigenomic analysis have been fundamental in providing a comprehensive insight into the mechanism of leukaemia progression and in identifying new molecular biomarkers for prognosis, disease control, and therapeutic stratification.

In this study, we have developed an unbiased bioinformatic pipeline to screen five independent patient gene expression arrays through which we show that higher expression of *WBP5* can serve to predict inferior outcome in patients with AML, thus providing a new potential stratification for therapy choices.

*WBP5* has been previously proposed as a candidate oncogene in colorectal cancers with microsatellite instability and its expression is associated with advanced gastric cancers with lymph node metastasis^18,19^. Moreover, Guo and co-workers were the first to report WBP5 expression to be significantly elevated in drug resistant small cell lung cancer patients and to be correlated with shorter survival time and advanced clinical stage. In this latter study, the authors showed that WBP5 modulates multidrug resistance both *in vitro* and *in vivo*, and that it does so by regulating the Hippo pathway^20,43^. Those studies reported that high expression of *WBP5* might be a predictor of unfavourable disease progression in many different cancers. Importantly, three independent studies provided a strong link between *WBP5* expression and adverse clinical outcome in leukaemia^21,22^. In fact, Metzeler and co-workers were the first to report *WBP5* as a risk factor by developing an 86-probe set gene expression signature to predict inferior outcome in patients with CN-AML. In this study, the authors showed patients that are characterized by an elevation of those genes displayed a strong association with FLT3-ITD mutation^21^. This work was followed by another study in which Bou Samra *et al*. proposed *WBP5* as part of a further refined prediction risk score that consisted of a panel of 22 genes, those being associated with poor prognosis in CN-AML patients. Importantly, the authors showed *WBP5* to rank first within this prediction panel according to the hazard ratio^22^. In line with those findings, our study demonstrates that high *WBP5* expressers have significantly lower overall and event-free survival and are associated with an unfavourable prognosis. We found that high *WBP5* expressers have a higher frequency of FLT3-ITD and NPM1c, which are often found together and are indicators of inferior outcome^44,45^. Conversely, we observed the *WBP5*^*low*^ sub-group to correlate with CEBPA and RUNX1-ETO lesions, which are generally associated with favourable outcome^46^. This is consistent with the reports that *WBP5* is associated with inferior survival in AML patients by displaying the highest hazard ratio and that its high expression correlates with advanced clinical stage and poor survival in lung cancers.

In order to gain an insight into the biological influence of aberrant *WBP5* expression in AML, we performed gene expression analysis in five independent patient expression arrays. Notably, we found that high *WBP5* levels correlated with elevated levels of several genes belonging to the HOXA and HOXB clusters; specifically, we observed a strong association with *HOXA9* and *HOXA10* and a moderate correlation with *HOXA7*. Many studies have demonstrated the importance of those genes in regulating the proliferation of haematopoietic cells and how their deregulation is paramount in driving the onset of myeloid leukaemias *in vivo*^47–50^. Importantly, we also observed a strong association of high *WBP5* levels with *HOXA9* oncogenic partners, among which *MEIS1*, *PBX3* and *FOXC1*^42,51,52^. We also observed elevated expression of a number of cancer-associated genes that have been reported to influence the proliferative advantage and the survival of leukaemic cells and genes that were reported to be part of the FLT3-ITD molecular signature, such as *CRNDE*, *CLU*, *CTSG*, *IGFBP2*, *CPA3* and *PRDM16*^34,35^. Notably, we also observed a strong association of *WBP5* with *WT1*, in agreement with previous reports. Our work suggests that a major mechanism of *WBP5* influencing leukaemia behaviour might directly or indirectly act through regulating these genes. To date, *WBP5* has been considered within risk prediction signatures to infer on clinical outcome but the importance of the sole *WBP5* expression as a reliable biomarker has not been investigated. We did so by performing survival analysis comparing low and high *WBP5* expressers in the cohort of patients from which those gene expression prognostic signatures have been determined and observed that *WBP5* alone was able to predict the same inferior clinical outcome in those patients. As such, our data suggest that it would be fruitful to perform a more elaborate study to further validate the importance of those findings *in vitro* and *in vivo*.

Taken together, our bioinformatic approach shows that *WBP5* expression is a reliable and powerful indicator of inferior outcome for AML and that it may be a candidate target for developing new therapeutic approaches.

## Supplementary information

Supplementary figures.

## Data Availability

The full code to reproduce the data presented in this manuscript is available at https://github.com/doncarlos999/WBP5_analysis.
