# Author Correction: High *WBP5* expression correlates with elevation of HOX genes levels and is associated with inferior survival in patients with acute myeloid leukaemia

**DOI:** 10.1038/s41598-020-70049-3

**Published:** 2020-07-30

**Authors:** C. Ward, P. Cauchy, P. Garcia, J. Frampton, M. A. Esteban, G. Volpe

**Affiliations:** 1 Key Laboratory of Regenerative Biology, Joint School of Life Sciences, Guangzhou Institutes of Biomedicine and Health, Chinese Academy of Sciences, Guangzhou, and Guangzhou Medical University, Guangzhou, China; 2 Laboratory of RNA, Chromatin and Human Disease, Guangzhou, 510530 China; 3 Key Laboratory of Regenerative Biology and Guangdong Provincial Key Laboratory of Stem Cell and Regenerative Medicine, Guangzhou Institutes of Biomedicine and Health, Chinese Academy of Sciences, Guangzhou, 510530 China; 4 Max Planck Institute of Immunobiology and Epigenetics, 79108 Freiburg, Germany; 5 Institute of Cancer and Genomic Sciences, College of Medical and Dental Sciences, University of Birmingham, Birmingham, B15 2TT UK; 6 Guangzhou Regenerative Medicine and Health Guangdong Laboratory, Guangzhou, 510005 China; 7 Institute for Stem Cells and Regeneration, Chinese Academy of Sciences, Beijing, 100101 China

Correction to: *Scientific Reports* 10.1038/s41598-020-60480-x, published online 26 February 2020

The original version of this Article contained an error in Affiliation 1, which was incorrectly given as

“Joint School of Life Sciences, Guangzhou Medical University and Guangzhou Institutes of Biomedicine and Health, Guangzhou, 511436, China.”

The correct affiliation is listed below:

Key Laboratory of Regenerative Biology, Joint School of Life Sciences, Guangzhou Institutes of Biomedicine and Health, Chinese Academy of Sciences, Guangzhou, and Guangzhou Medical University, Guangzhou, China.

This error has now been corrected in the HTML and PDF versions of the Article.

