# Supplementary material for: High WBP5 expression correlates with elevation of HOX genes levels and is associated with inferior survival in patients with acute myeloid leukaemia
Source: Sci Rep. 2020 Feb 26;10:3505. doi: 10.1038/s41598-020-60480-x (PMC7044279; doi:10.1038/s41598-020-60480-x)
Supplement: Supplementary file 1 — Supplementary figures. [file 41598_2020_60480_MOESM1_ESM.pdf]

High *WBP5* expression correlates with elevation of HOX genes levels and is associated with inferior survival in patients with acute myeloid leukaemia

Ward C, Cauchy P, Garcia P, Frampton J, Esteban MA and Volpe G

Supplementary information

Figure S1. Validation of survival analysis in GSE12417 and GSE37642 datasets

Kaplan-Meier representation of overall survival (OS) for *WBP5*^{low} and *WBP5*^{high} AML patients from the GSE12417[21] (left panel) and GSE37642[29] (right panel) prior to or after balancing for age, sex, FAB and cytogenetic findings. The number of patients and the median OS are indicated in the plot. P-value was calculated using log rank Mantel-Cox t-test.

Figure S2. Hierarchical clustering of GSE1159, GSE22845 and GSE13204 datasets.

Hierarchical clustering of the Pearson correlation coefficient of mRNA values showing distinct clusters for high and low *WBP5* expressers from the Valk (GSE1159), Haferlach (13204) and Taskesen (GSE22845) cohorts. The mutational status of every patient is indicated by a colour coded graph on the top of the clustering map. A legend indicating each genetic subgroup is placed below the clustering map.

Figure S3. HOXA and HOXB clusters gene expression in all patient cohorts.

Expression of all HOXA and HOXB cluster genes, HOX partners (*MEIS1, MEIS2, MEIS3*, *PBX1*, *PBX2* and *PBX3*) and *WT1*. Data are represented as boxplots in which high expressers are indicated in red and low expressers are indicated in blue. Every plot presents a color-coded boxplot showing a median interquartile range.

Figure S4. Correlation of FLT3-ITD signature genes expression with high *WBP5* levels in AML patients.

Boxplots indicating the expression of representative FLT3-ITD molecular signature genes (*CRNDE*, *CLU*, *CTSG*, *IGFBP2*, *CPA3*, *PRDM16*) comparing *WBP5*^{low} and *WBP5*^{high} AML patients from the Verhaak (GSE6891), Kohlmann (GSE15434), Valk (GSE1159), Haferlach (GSE13204) and Taskesen (GSE22845) cohorts. Every plot presents a color-coded boxplot showing a median interquartile range.

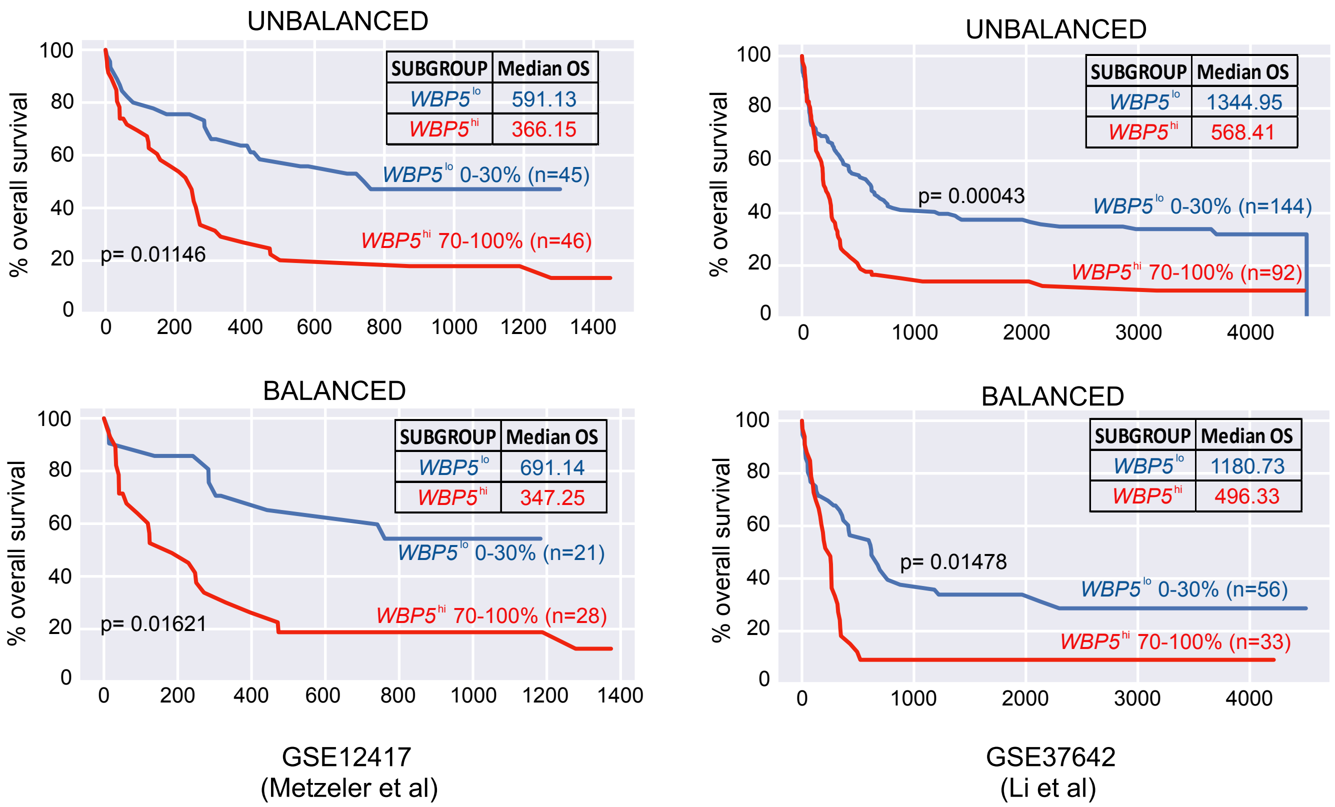
UNBALANCED
% overall survival
SUBGROUP | Median OS
WBP5lo | 591.13
WBP5hi | 366.15
WBP5lo 0-30% (n=45)
WBP5hi 70-100% (n=46)
p= 0.01146
UNBALANCED
% overall survival
SUBGROUP | Median OS
WBP5lo | 1344.95
WBP5hi | 568.41
p= 0.00043
WBP5lo 0-30% (n=144)
WBP5hi 70-100% (n=92)
BALANCED
% overall survival
SUBGROUP | Median OS
WBP5lo | 691.14
WBP5hi | 347.25
WBP5lo 0-30% (n=21)
WBP5hi 70-100% (n=28)
p= 0.01621
BALANCED
% overall survival
SUBGROUP | Median OS
WBP5lo | 1180.73
WBP5hi | 496.33
p= 0.01478
WBP5lo 0-30% (n=56)
WBP5hi 70-100% (n=33)
GSE12417
(Metzeler et al)
GSE37642
(Li et al)

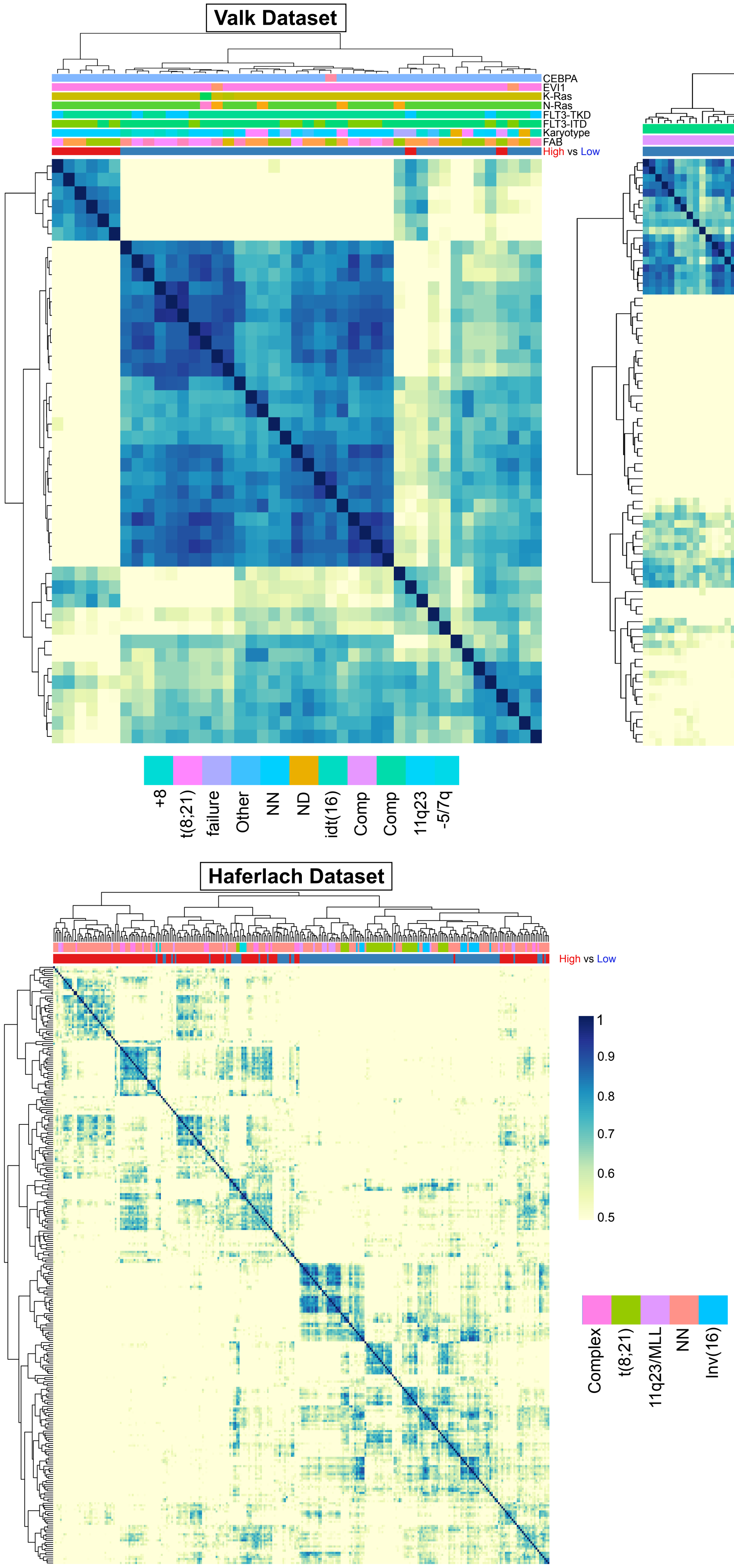
Valk Dataset
CEBPA
EVI1
K-Ras
N-Ras
FLT3-TKD
FLT3-ITD
Karyotype
FAB
High vs Low
+8
t(8;21)
failure
Other
NN
ND
idt(16)
Comp
Comp
11q23
-5/7q
Taskesen Dataset
High vs Low
C/EBPα status
WT
Mono-allelic
Bi-allelic
Karyotype
AML 46,XY,9ph
AML 46,XY,inv(9)(p11q13)c
AML 46,XY
Haferlach Dataset
High vs Low
1
0.9
0.8
0.7
0.6
0.5
Complex
t(8;21)
11q23/MLL
NN
Inv(16)

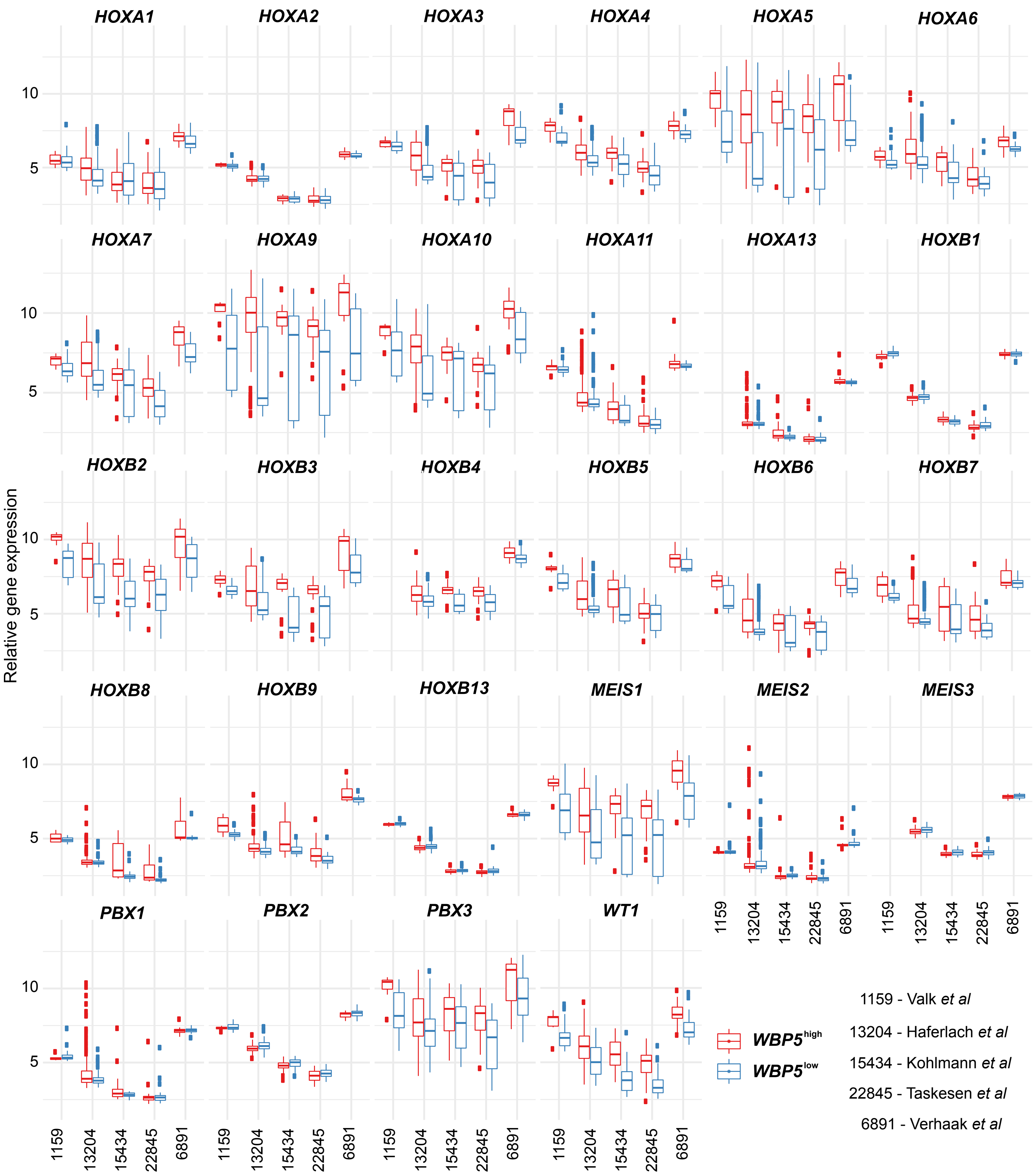

HOXA1
HOXA2
HOXA3
HOXA4
HOXA5
HOXA6
HOXA7
HOXA9
HOXA10
HOXA11
HOXA13
HOXB1
HOXB2
HOXB3
HOXB4
HOXB5
HOXB6
HOXB7
HOXB8
HOXB9
HOXB13
MEIS1
MEIS2
MEIS3
PBX1
PBX2
PBX3
WT1
Relative gene expression
10
5
1159
13204
15434
22845
6891
WBP5high
WBP5low
1159 - Valk et al
13204 - Haferlach et al
15434 - Kohlmann et al
22845 - Taskesen et al
6891 - Verhaak et al

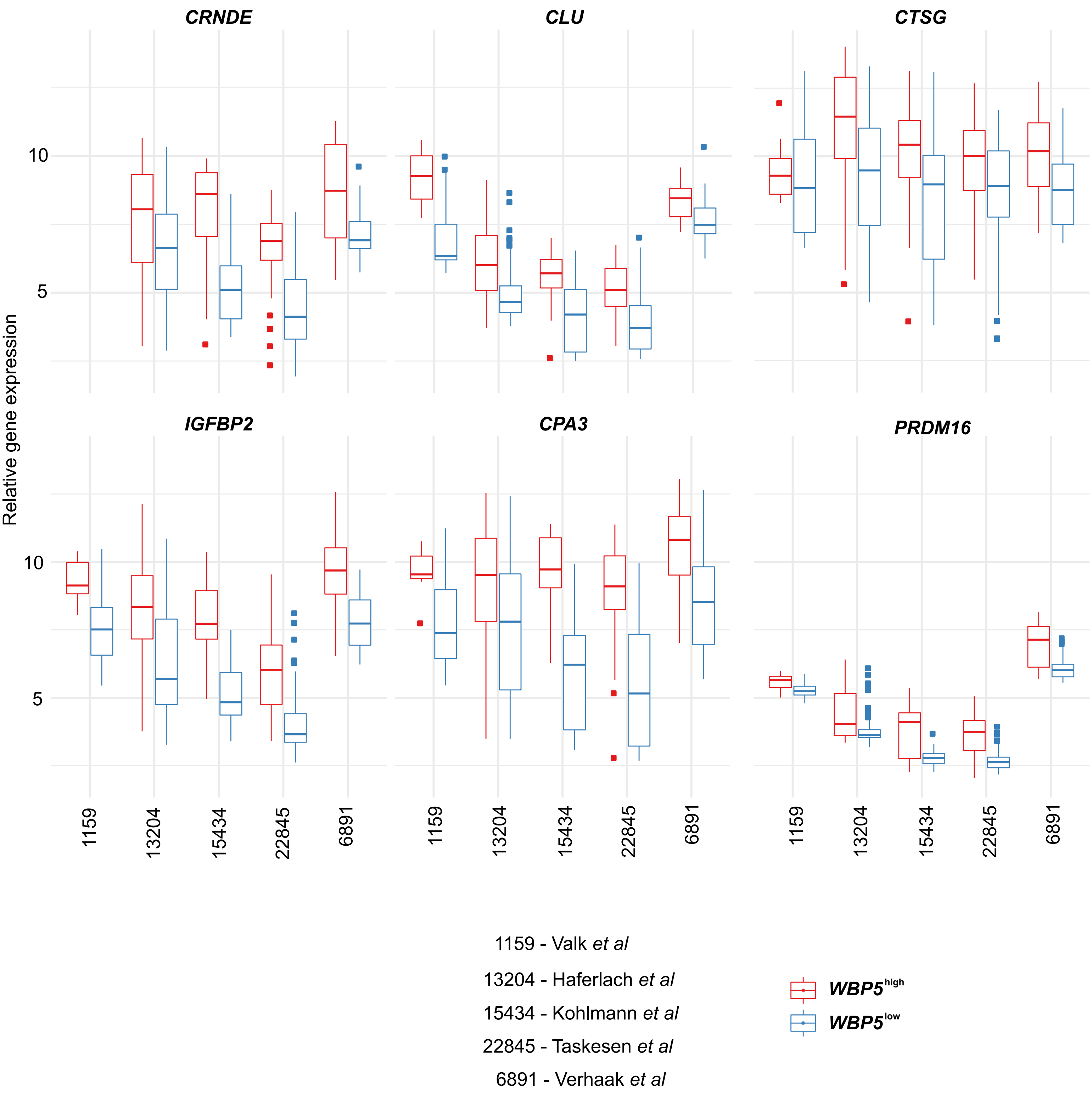

CRNDE
CLU
CTSG
IGFBP2
CPA3
PRDM16
Relative gene expression
10
5
1159
13204
15434
22845
6891
1159 - Valk *et al*
13204 - Haferlach *et al*
15434 - Kohlmann *et al*
22845 - Taskesen *et al*
6891 - Verhaak *et al*
*WBP5*high
*WBP5*low
